# High resolution, high speed, long working distance, large field of view confocal fluorescence microscope

**DOI:** 10.1038/s41598-017-13778-2

**Published:** 2017-10-17

**Authors:** Shaun Pacheco, Chengliang Wang, Monica K. Chawla, Minhkhoi Nguyen, Brend K. Baggett, Urs Utzinger, Carol A. Barnes, Rongguang Liang

**Affiliations:** 1 College of Optical Sciences, University of Arizona, Tucson, Arizona 85721 USA; 2 School of Information Engineering, Wuhan University of Technology, Wuhan, Hubei 430070 China; 3 Evelyn F. McKnight Brain Institute, University of Arizona, Tucson, Arizona 85721 USA; 4 ARL Division of Neural Systems, University of Arizona, Tucson, Arizona 85721 USA; 5 Department of Biomedical Engineering, University of Arizona, Tucson, Arizona 85721 USA; 6 Departments of Psychology, Neurology and Neuroscience, University of Arizona, Tucson, Arizona 85721 USA

## Abstract

Confocal fluorescence microscopy is often used in brain imaging experiments, however conventional confocal microscopes are limited in their field of view, working distance, and speed for high resolution imaging. We report here the development of a novel high resolution, high speed, long working distance, and large field of view confocal fluorescence microscope (H^2^L^2^-CFM) with the capability of multi-region and multifocal imaging. To demonstrate the concept, a 0.5 numerical aperture (NA) confocal fluorescence microscope is prototyped with a 3 mm × 3 mm field of view and 12 mm working distance, an array of 9 beams is scanned over the field of view in 9 different regions to speed up the acquisition time by a factor of 9. We test this custom designed confocal fluorescence microscope for future use with brain clarification methods to image large volumes of the brain at subcellular resolution. This multi-region and multi-spot imaging method can be used in other imaging modalities, such as multiphoton microscopes, and the field of view can be extended well beyond 12 mm × 12 mm.

## Introduction

Reconstructing wiring diagrams in the brain still remains a significant challenge for neuroscientists. The ability to identify the specific networks involved in controlled behaviors could lead to the discovery of critical circuit differences that differentiate normal versus abnormal brain function. A fundamental understanding of these differences could potentially lead to effective approaches to treat, prevent, or cure brain disorders like Alzheimer’s disease, schizophrenia, autism, epilepsy, and traumatic brain injury.

One common method used to reconstruct wiring diagrams is to cut the whole brain into smaller sections^1–3^. The sectioned tissue has to be thin enough for optical microscopes to image the entire volume without degrading the image quality significantly. Light scatter in the tissue is the primary factor that limits the penetration depth of microscopes. The optical sectioning capabilities of conventional confocal fluorescence microscopes allows good image quality up to a couple hundred microns into the tissue. Therefore, the tissue sections have to be less than a hundred microns thick to obtain good image quality with these microscopes. While a two-photon microscope penetrates deeper than a confocal microscope, the penetration depth is still limited. In order to achieve whole brain imaging using two-photon microscopes, the brain still has to be sectioned^3^.

Not only is the process of fully sectioning a brain time-consuming, but there is also an inherent disadvantage in studying the brain using this method. During sectioning, pathways in the brain may become severed, leading to difficulty in accurately aligning pathways between two adjacent sections. Furthermore, aligning long-range pathways over several millimeters in depth becomes extremely difficult. Therefore, it is challenging to fully understand the 3D structure of the brain using sectioned tissue. While understanding these wiring diagrams at the microscopic level is important, to fully understand the brain, the macro and microscopic data need to be merged for a system level understanding of the function of the brain^4^.

In recent years, several brain clarification methods, such as CLARITY^5^, CUBIC^6^, BABB^7^, and Scale^8^, have been demonstrated. Brain clarification methods are processes in which the brain’s structural and molecular information is retained, but the brain is rendered optically transparent and macro-molecule permeable. Light scattering is significantly reduced using brain clarification techniques, permitting conventional fluorescence microscopes to penetrate much deeper into the brain.

Even though the brain clarification methods solve the problem of light penetration depth for brain imaging, microscopes are typically not optimized for whole brain imaging. As the numerical aperture (NA) of the objective increases, the field of view (FOV) and the working distance decrease. Therefore, high NA objectives often have short working distances, which makes it impossible to image an entire non-sectioned brain sample, because the sample will hit the surface of the objective before it can image through to the bottom of the brain. For example, the microscope used by Chung *et al*.^5^ to image a 5–6 mm thick mouse brain using single photon excitation, had a working distance of 3.6 mm. To image the whole brain, first the dorsal half of the brain was imaged, then the brain was inverted and the ventral half was imaged. Long working distance objectives have been made; however, the FOV is typically small. Because of this the brain would have to be moved sequentially across the FOV of the objective to cover the entire region of the brain.

As an example, for an objective with a FOV of 0.5 mm × 0.5 mm and with the area of interest being 11 mm × 11 mm, the sample needs to be translated 484 times, assuming no overlap between each successive image, to cover the entire extent of the brain. For an objective designed with a FOV of 6 mm × 6 mm, however, the objective only needs to be translated 4 times to cover the same 11 mm × 11 mm region. Therefore, it is extremely advantageous to have a larger FOV to significantly speed up brain imaging. This problem is compounded even further, since at every lateral position across the brain, the brain is also scanned axially. Suppose at every position there are *N*_*z*_ axial movements, then the brain with the smaller FOV has to be translated 484*N*_*z*_ times, whereas the larger FOV only has to be translated 4*N*_*z*_ times. Therefore, the larger FOV has the potential to significantly reduce the total acquisition time for whole brain imaging.

Recently, large FOV two-photon microscopes have been demonstrated to overcome the traditionally small FOVs of two-photon microscopes^9,10^. Susaki *et al*.^6^ used two independently positionable, temporally multiplexed excitation pathways to speed up imaging two regions of the brain simultaneously. Sofroniew *et al*.^10^ developed a 2-photon random access mesoscope (2p-RAM) that allows high resolution imaging anywhere within a volume spanning multiple brain areas. Both approaches increase the FOV, but the scanning speed is still limited by a single-point scanning method.

While this paper discusses the application of the proposed concept in brain imaging, the same concept can be applied in other fields, such as whole slide scanner and semiconductor inspection.

## Concept of a high resolution, high speed, long working distance, and large field of view confocal fluorescence microscope

**Figure 1:**
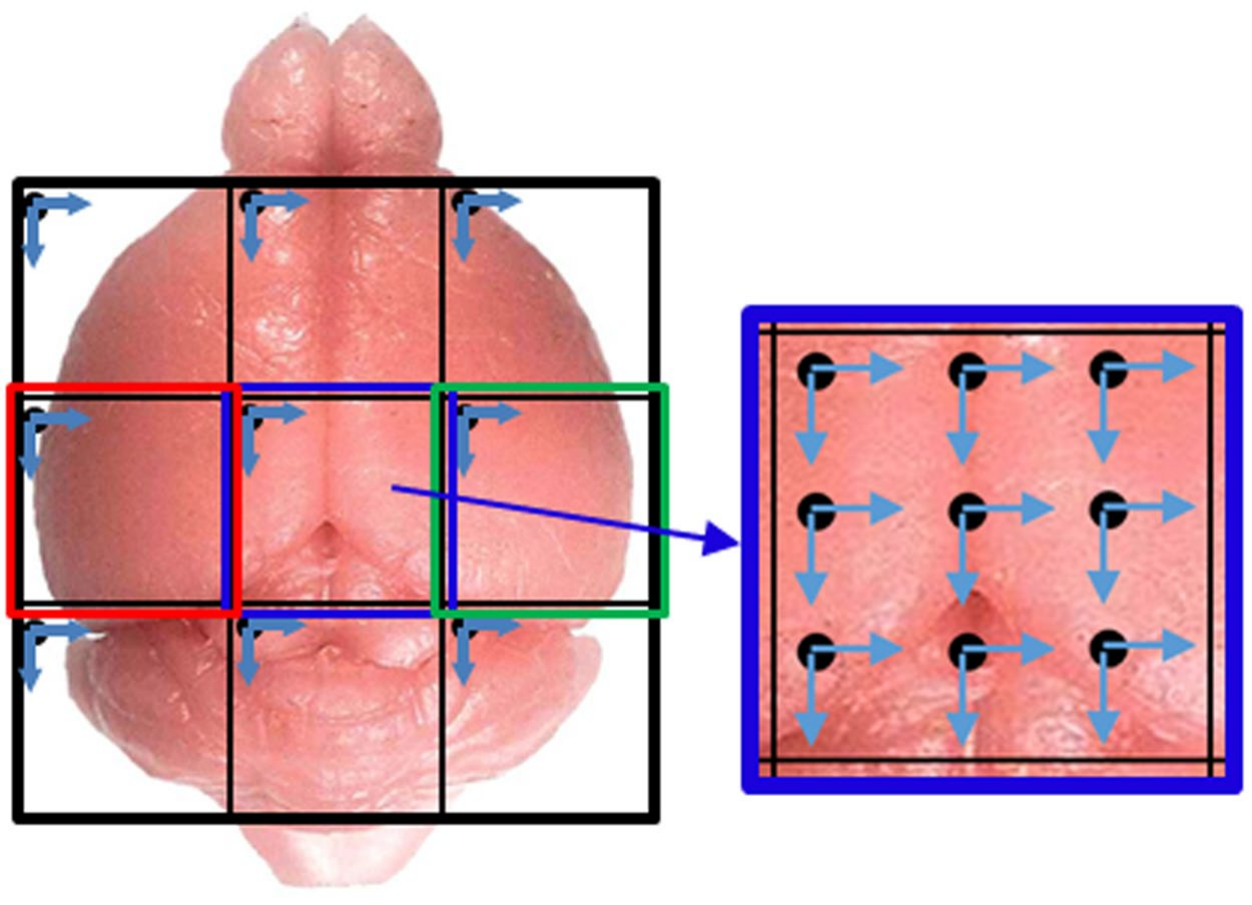
Concept of multi-region and multifocal scanning microscope

We have developed a prototype of a novel high resolution, high speed, long working distance, and large FOV confocal fluorescence microscope (H^2^L^2^-CFM). To improve the imaging speed, we use a multi-region and multifocal scanning method as shown in Fig. 1. This method divides the entire FOV into sub-regions (3 × 3, 4 × 4, 5 × 5, etc.) with each region having its own excitation and imaging channel. All channels will share the scanner, relay lens, and objective. With the multichannel scanning approach proposed, imaging speed is increased significantly compared to single channel methods. For example, for a 3 × 3 channel configuration, the scanning speed is ~9x faster. To further improve the scanning speed, we could develop multifocal scanning methods combined with multiple channels as shown in Fig. 1 (for example, sub-region and its related imaging channel in blue). When the multifocal scanning method is used in each channel, the imaging speed will be further increased by another factor of the number of focal points. For example, when the 3 × 3 multifocal point scanning is used in each of 3 × 3 channels, the imaging speed is ~(3 × 3)x(3 × 3) = ~81x faster than a conventional single point multiphoton microscope. Potentially we can develop a confocal fluorescence or multiphoton microscope with a FOV > 12 mm × 12 mm and the number of scanning spots >225. To demonstrate the concept, we prototype a 0.5 NA system with 3 mm × 3 mm field of view and 12 mm working distance. To speed up the acquisition time, an array of 9 beams are used to scan the FOV 3 mm × 3 mm to reduce the scanning time over that FOV by a factor of 9. The microscope objective is specifically designed for whole brain imaging with a large FOV and a long working distance to image cleared rat brains, so the whole rat brain can be imaged without flipping or sectioning.

## System design

### System layout

**Figure 2:**
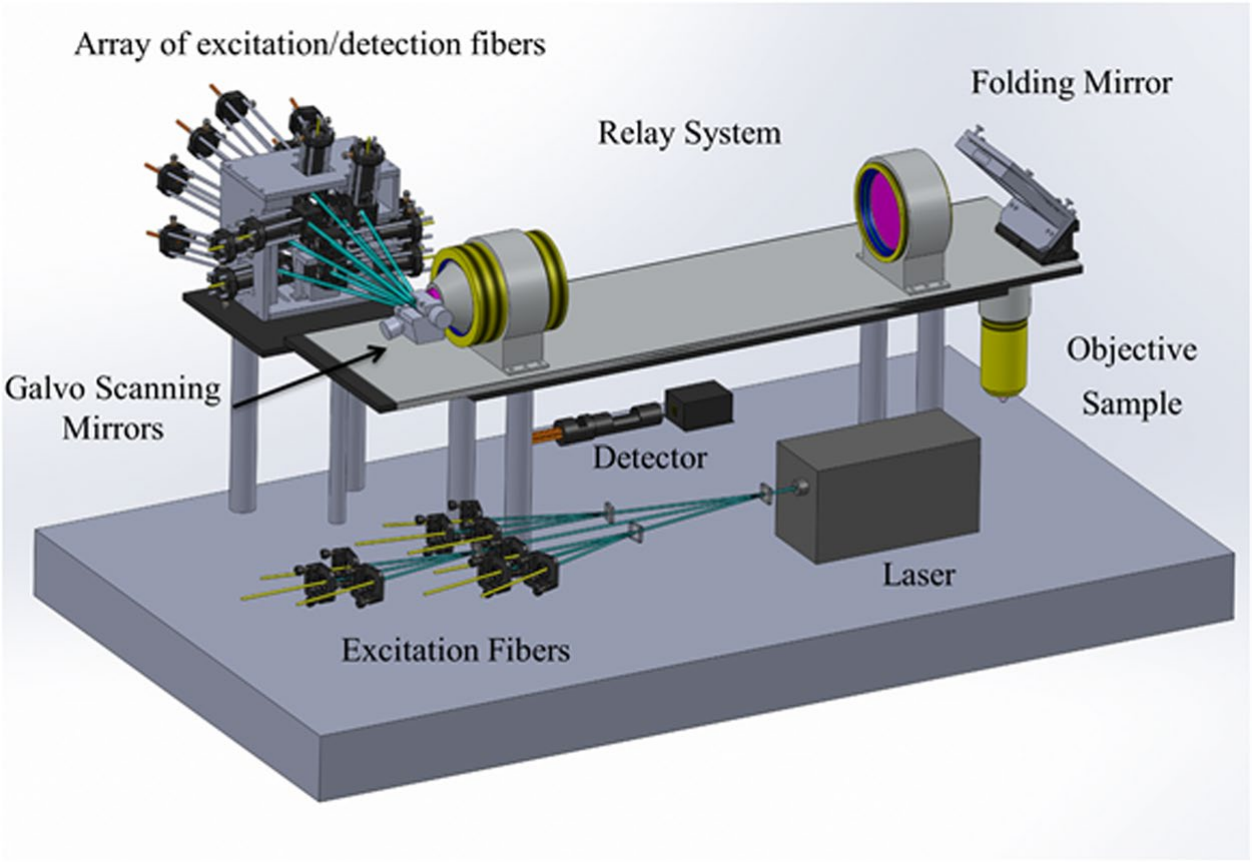
Layout of the H^2^L^2^-CFM.

Figure 2 is the layout of the prototyped confocal fluorescence microscope, it has a 0.5 NA objective, a 3 mm × 3 mm FOV, and 12 mm working distance. The excitation wavelength is 488 nm, and the optical systems are optimized for a wavelength range of 480 nm – 580 nm. A summary of the design is as follows: a 488 nm argon-ion laser is split into 9 equal energy beams using beam-splitting gratings. Each of these 9 beams is coupled into a single-mode fiber. A mechanical mount is designed, such that the light from each single-mode fiber is collimated and is reflected off a dichroic mirror, where each beam converges onto a galvo-scanning mirror at a specified angle. The angle between each channel determines the relative position of focal spots in the focal plane. 3 × 3 channels are aligned so that 3 × 3 channels can cover the 3 × 3 mm FOV with 0.05 mm overlap. A relay system relays the beams into the entrance pupil of the objective, and the 9 beams are focused at the sample plane, where the 9 beams are spatially separated in a 3 × 3 array. The sample is excited by the 9 beams at different spatial positions, and the fluorescent light travels back through the objective and relay system, traversing the reverse path of the excitation light. The florescence transmits through a dichroic mirror, where the fluorescent light is focused onto the end of a multi-mode fiber. The light exiting from each multi-mode fiber is relayed onto a PMT array, where the intensity of the fluorescent signal is measured and transferred to a computer. Each component of the microscope is detailed in the following sub-sections.

### Coupling to excitation fibers

**Figure 3:**
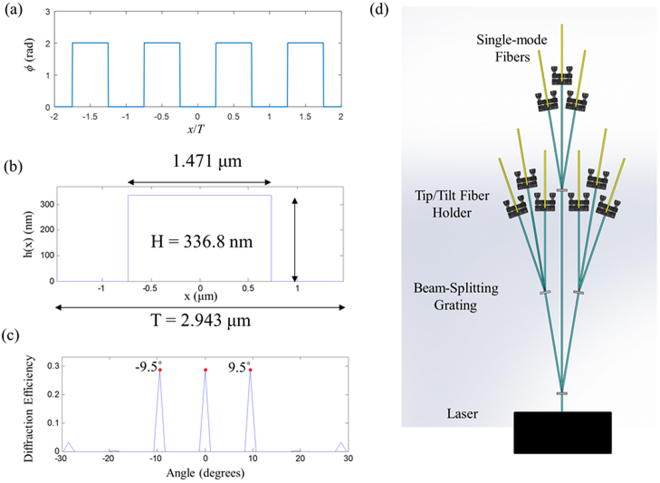
(**a**) Phase profile for 1 × 3 Dammann grating. (**b**) Height profile for fabricated grating. (**c**) Far-field diffraction pattern. (**d**) Layout for single-mode fiber coupling for 9 beams.

A 1 × 3 binary Dammann grating is used as the beam-splitting optics for this microscope^11^. The Dammann grating has a phase *ϕ*_0_ = 2.008 radians and the phase profile is shown in Fig. 3(a). The height of the grating is calculated using

1 $$h=\frac{{\varphi }_{0}}{2\pi }\frac{\lambda }{n(\lambda )-1}$$

where *λ* is the wavelength, and *n* is the index of refraction at wavelength *λ*. For this microscope *λ* = 488 nm and the Dammann grating is made from fused silica. The index of fused silica is *n* = 1.4630 at *λ* = 488 nm. The grating is designed to have a diffraction angle of 9.5°. The designed height profile is shown in Fig. 3(b) and the resulting diffraction pattern of the Dammann grating is shown in Fig. 3(c). Note the diffraction pattern consists of three equal energy beams diffracted at an angle of 9.5°.

The layout to split the incident laser beam into 9 outgoing beams is shown in Fig. 3(d). Collimated light from a laser is incident on a Dammann grating, which splits the beam into 3 equal energy collimated output beams. Each of these three beams travels for some distance and is then incident on another Dammann grating. Therefore, each of these three beams is split into three more beams. An adjustable aspheric collimator is mounted on a tip/tilt stage and the light from each of the 9 beams is coupled into a single-mode fiber. Ignoring absorption losses and Fresnel reflections, theoretically 7.8% of the incident light reaches the single-mode fiber based on diffraction efficiency. Experimentally, 5% ± 1% of the incident light is transmitted through each fiber.

### Array of confocal arms

**Figure 4:**
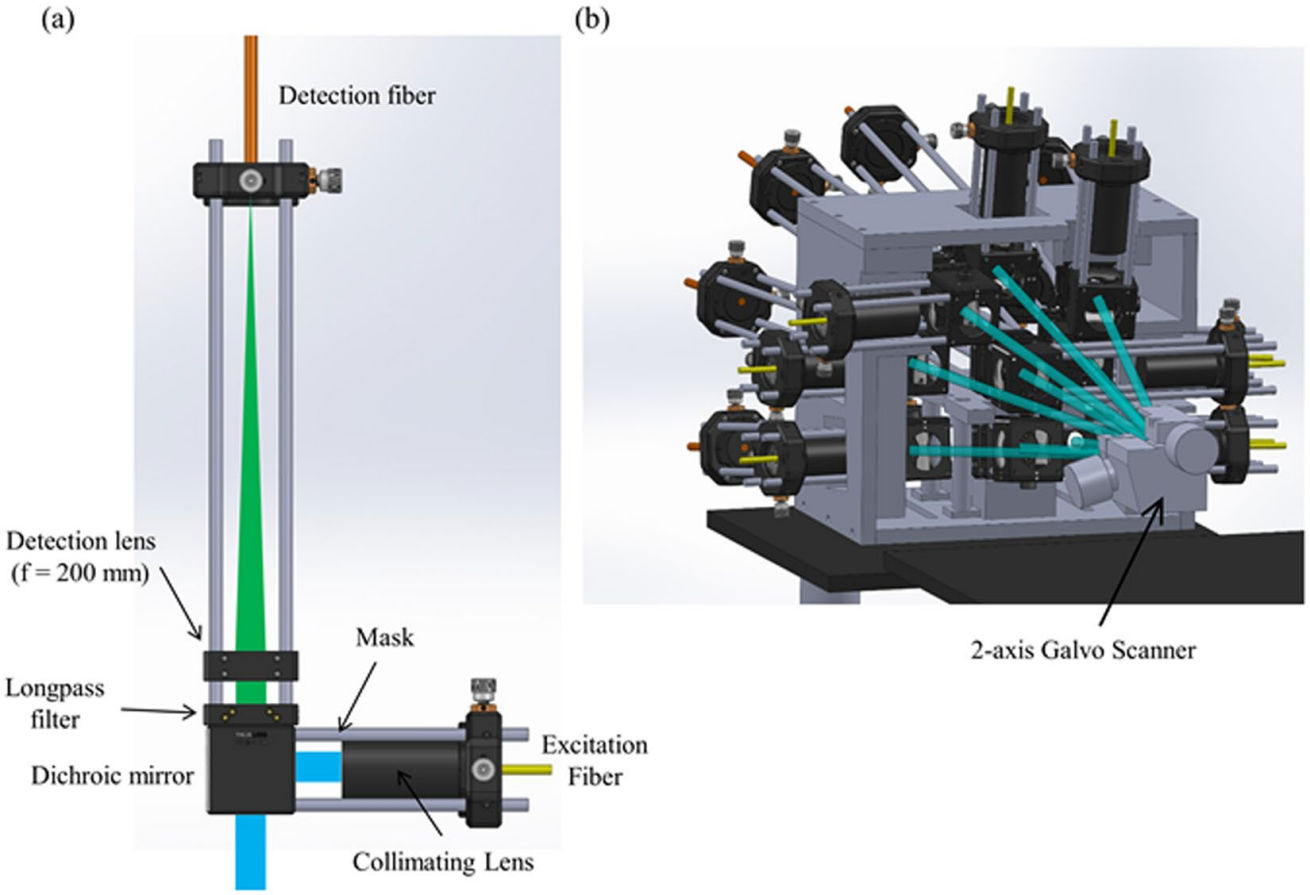
(**a**) Layout of the confocal arm. (**b**) Mechanical mount to hold array of confocal arms.

The other end of each of the 9 single-mode fibers is attached to a confocal arm as shown in Fig. 4(a). The excitation fiber acts as the illumination pinhole for the confocal microscope and a multimode fiber acts as the detection pinhole. The light from the single-mode fiber is collimated using a 30 mm lens positioned inside a lens tube and is clipped to a diameter of 7 mm using a mask. The beam reflects off a dichroic mirror and is directed to reflect off a 2-axis galvo scanner.

In order for the beams to be separated by 1 mm in the *x* and *y* direction at the sample plane, the beams need to be separated by an angle of 9° when incident on the galvo scanner. A mechanical mount, as shown in Fig. 4(b), angles each of the confocal arms to create the required angular separation between each of the 9 beams. The sides of the mechanical mount are tilted, such that each confocal arm sends the excitation light at the required angle.

### Relay system and objective

**Figure 5:**
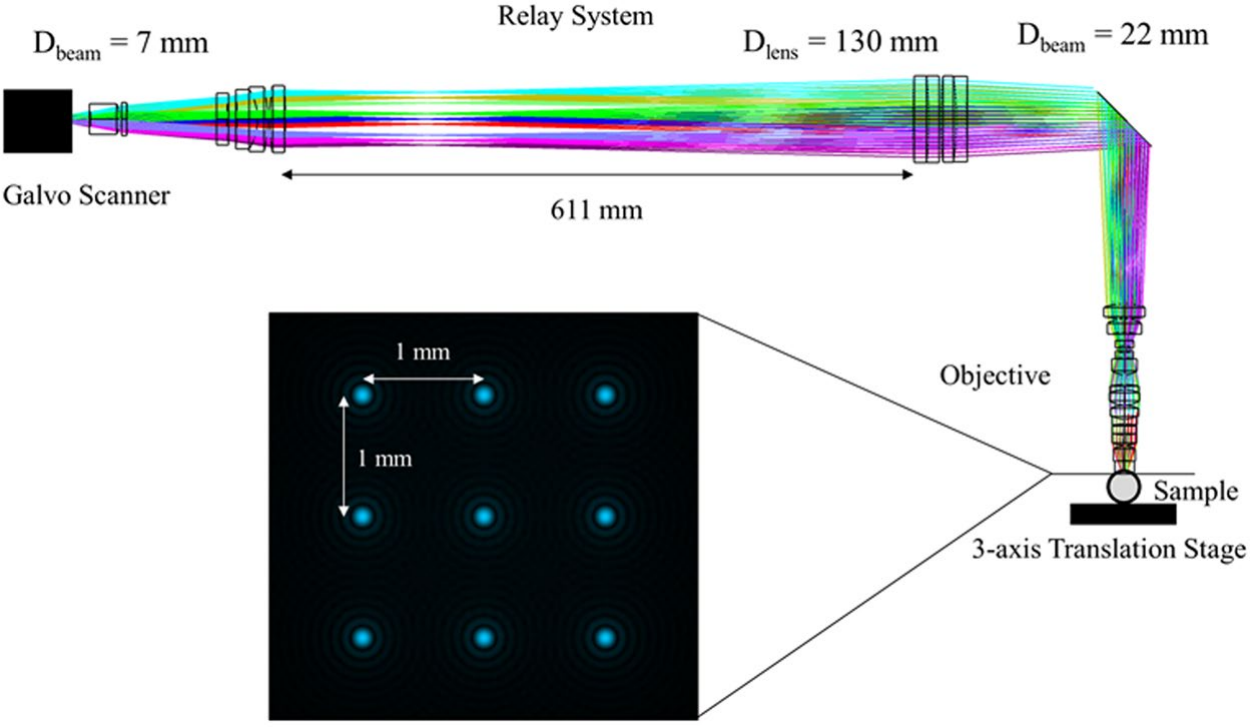
Optical layout for relay system and objective.

The optical design of the relay system and objective is shown in Fig. 5. The beams are separated by 9° after the galvo system. Through the relay system, the beams are enlarged from a diameter of 7 mm to a diameter of 22 mm after the galvo scanning mirrors. All 9 beams are relayed to the entrance pupil of the objective, where all 9 beams are then focused onto a 3 × 3 array at the sample plane, as shown in Fig. 5.

**Figure 6:**
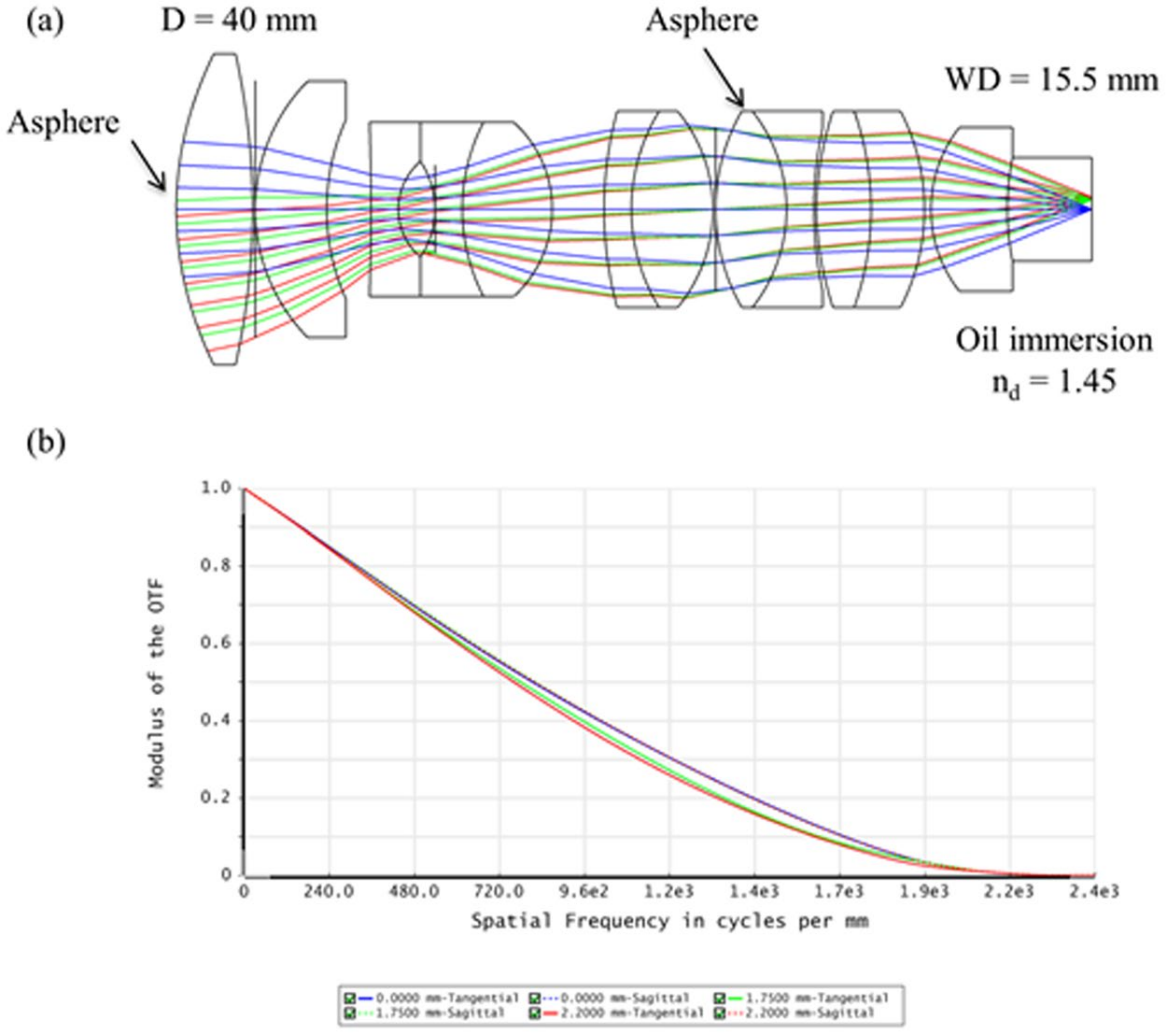
(**a**) Optical design for microscope objective. (**b**) MTF for objective, showing near diffraction-limited performance over the entire FOV.

The optical design for the microscope objective is shown in Fig. 6(a). The objective is designed to have a 0.5 NA and is designed to image a 3 mm × 3 mm FOV with a 12 mm working distance. It’s an oil immersion objective, designed for oil with an index of *n*_*d*_ = 1.45. The objective is optimized for the wavelengths from 480 nm – 580 nm. As shown in Fig. 6(b), the MTF shows the diffraction limited performance over the entire FOV.

### Detection

The fluorescence from each of the 9 beams travels back through the objective and relay system. After reflecting off of the galvo scanning mirror, the fluorescence from each beam returns to its respective confocal arm. The fluorescent light is transmitted through a dichroic mirror and is transmitted through a longpass filter that transmits wavelengths greater than 500 nm. A 200 mm lens is used to focus the fluorescence onto the end of a multimode fiber, which acts as the detection pinhole, as shown in Fig. 4(a). Assuming a 12.5 mm diameter aperture, after focusing the 200 mm lens, the polychromatic Airy disk radius is 9.37 μm over the wavelength range 480 nm – 580 nm. Due to the limited choices of fiber diameters and off-the-shelf focusing lenses, we use a multimode fiber with a core diameter 25 μm, the fiber core diameter is 1.33 times the Airy diameter.

## System performance

**Figure 7:**
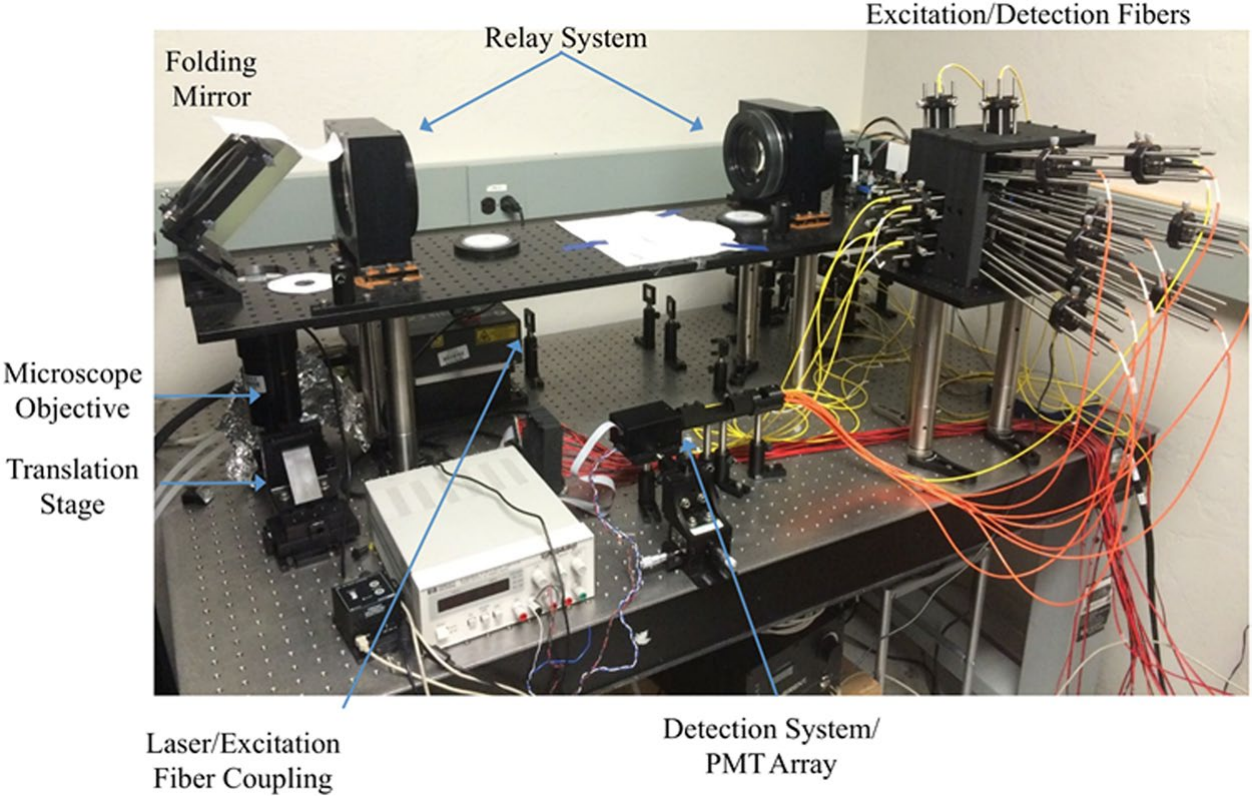
Photograph of built H^2^L^2^-CFM with components labeled.

Figure 7 is the photograph of the prototyped H^2^L^2^-CFM, the detection electronics is not shown in the figure.

### Working distance

**Figure 8:**
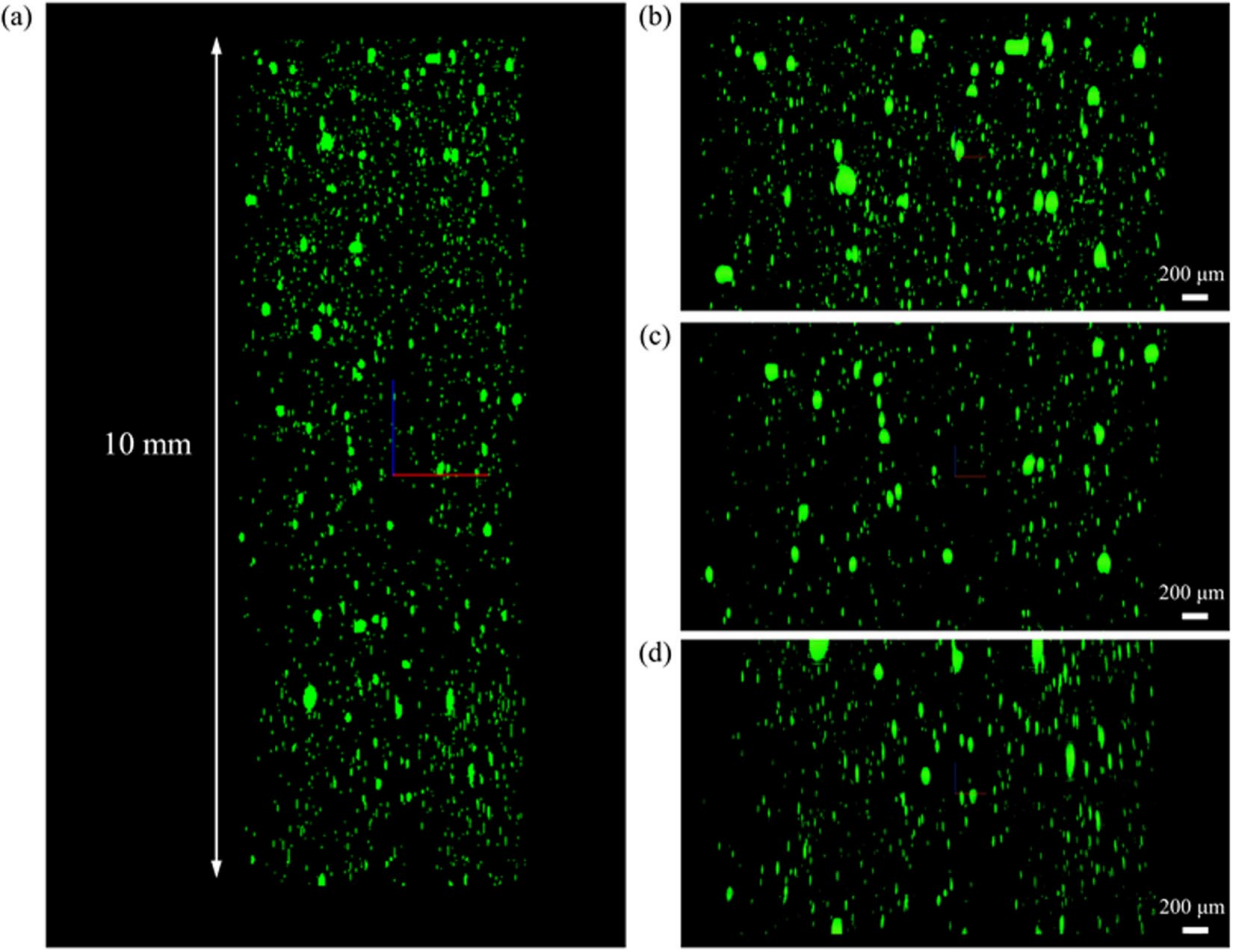
(**a**) xz-view of volume of fluorescent microspheres. Magnified views of (**b**) top, (**c**) middle, and (**d**) bottom regions of the *xz*-view.

To test the working distance, 0.5 μm diameter fluorescent microspheres are suspended in polydimethylsiloxane (PDMS), with an index of refraction of 1.4, which is close to the designed index of 1.45 for the objective. The cylindrical volume of fluorescent microspheres is placed in a container full of oil immersion liquid with an index of 1.45. The volume is scanned 10 mm in depth with 3 μm axial spacing between consecutive images. The volume is moved axially by a translation stage. Microspheres are visible and distributed throughout all 9 channels. In some regions the volume of the microspheres did not disperse completely and the microspheres clumped together, which explains the larger objects in Fig. 8. Figure 8(a) shows the *xz*-view and Fig. 8(b–d) show magnified *xz*-views in the top, middle, and bottom of the volume, respectively. The fluorescent microspheres are visible throughout the entire 10 mm, demonstrating the working distance of the objective is at least 10 mm.

### Resolution

**Figure 9:**
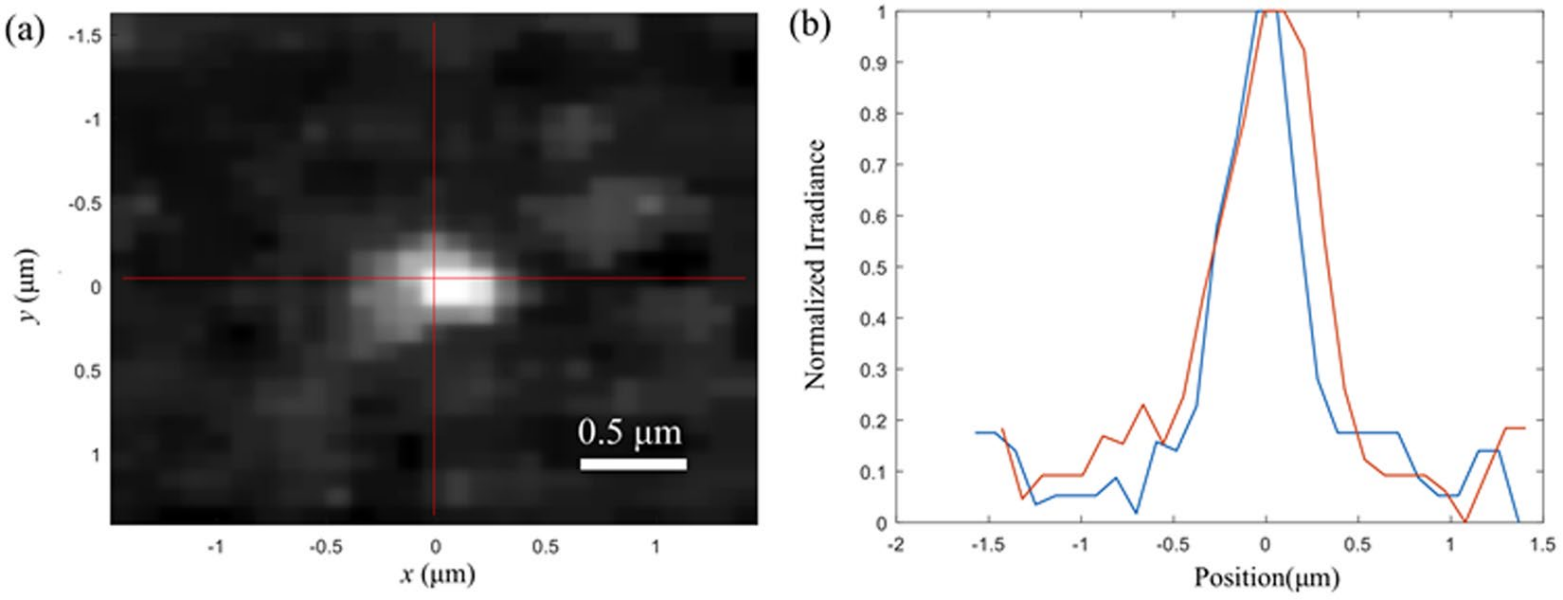
(**a**) Image of 200 nm fluorescent microsphere, which approximates the PSF. (**b**) A cross-section through lines displayed in (**a**).

The resolution of the system is estimated by measuring the point spread function (PSF) in each of the 9 channels. The PSF is estimated by imaging 200 nm fluorescent microspheres^12^. The fluorescent microspheres are placed on a microscope slide and covered with a cover glass. The microscope slide is then placed in a container of oil immersion liquid. A cropped median-filtered image of one microsphere is shown in Fig. 9(a). A Gaussian function is fit to the image of the microsphere, which is used to calculate the full width half maximum (FWHM) of the PSF. A cross-section of the bead is shown in the horizontal and vertical directions in Fig. 9(b). The average FWHM for all 9 channels is 555 nm ± 88 nm. In practice, confocal microscopes give little lateral resolution improvement over bright field microscopy due to a number of practical considerations^13^. The detection pinhole in this system is 1.33 times the Airy disk radius, which is going to cause deviation from the confocal resolution. Also, the cover glass from the microscope slide introduces spherical aberration into the system, which also reduces the resolution. Due to the complexity of this system, some misalignment between elements may also exist, which also reduces the resolution by introducing aberration into the system.

### Neuroscience applications for the H^2^L^2^-CFM microscope

**Figure 10:**
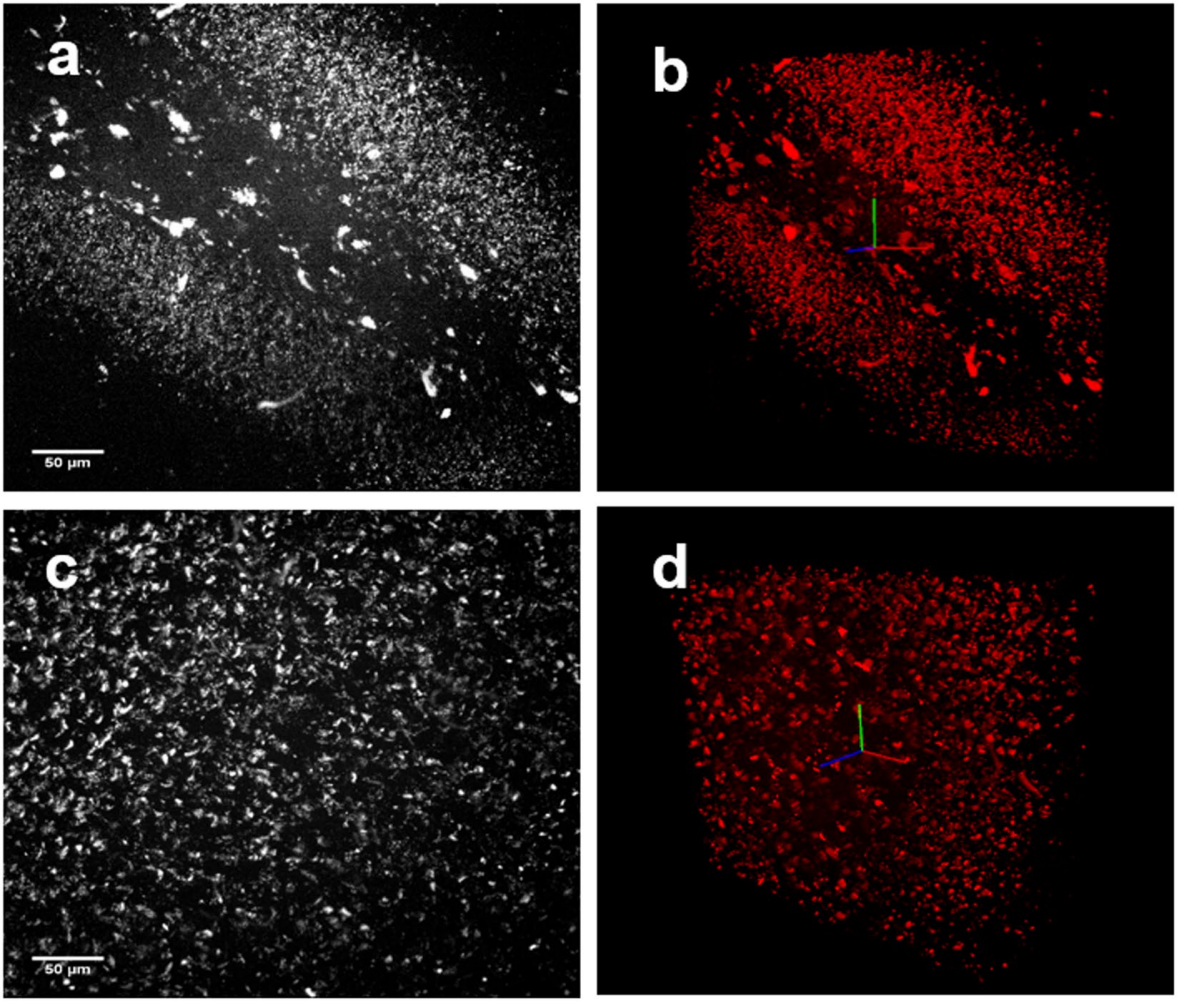
Fluorescence *in situ* hybridization for *Arc* mRNA was performed on a cleared rat brain. The brain was cut into 1 mm coronal sections, mounted in an agarose block and immersed in 85% glycerol for imaging using a multi-photon microscope. (**a**) shows the maximum projection image of the dentate gyrus tip of the hippocampus in the rat temporal lobe, imaged over a depth of 225 μm with a 3 μm step size. (**b**) is a 3D reconstruction of the hippocampus image shown in (**a**) where the upper and lower blade of the dentate gyrus and hilus have visible *Arc*-positive labeling (red). (**c**) shows the maximum projection image taken of the rat parietal cortex over a depth of 705 μm imaged in increments of 5 μm. (**d**) is a 3D reconstruction of the image from cortex shown in (**c**).

One major use of this instrument will be to enable visualization of behavior-driven circuit activity, with the ultimate goal being to image an entire brain. One critical component to achieving whole brain activity mapping is to refine the tools available for macromolecular labelling of active cells. To this end we have begun to optimize the coupling of tissue clearing methods combined with amplification of fluorescence labelling probes using hybridization chain reaction techniques. To detect mRNA targets, hybridization chain reaction utilizes short DNA probes, which offers increased diffusion and penetration of tissue samples^14^. In addition, an orthogonal amplification of the fluorescence signal by short DNA hairpin polymerization of the RNA-DNA hybrid scaffold leads to more efficient labeling in larger tissue volumes. Fluorescence *in situ* hybridization (FISH) of the immediate early gene *Arc* was used to image a cleared rat brain using a conventional 2 photon microscope. Figure 10(a and b) show an image of tissue containing the hippocampus region of the rat brain following the FISH procedure, which was imaged to a depth of 225 μm at a 3 μm step size. Figure 10(c and d) contains images of the rat parietal cortex obtained over a depth of 705 μm in increments of 5 μm steps.

**Figure 11:**
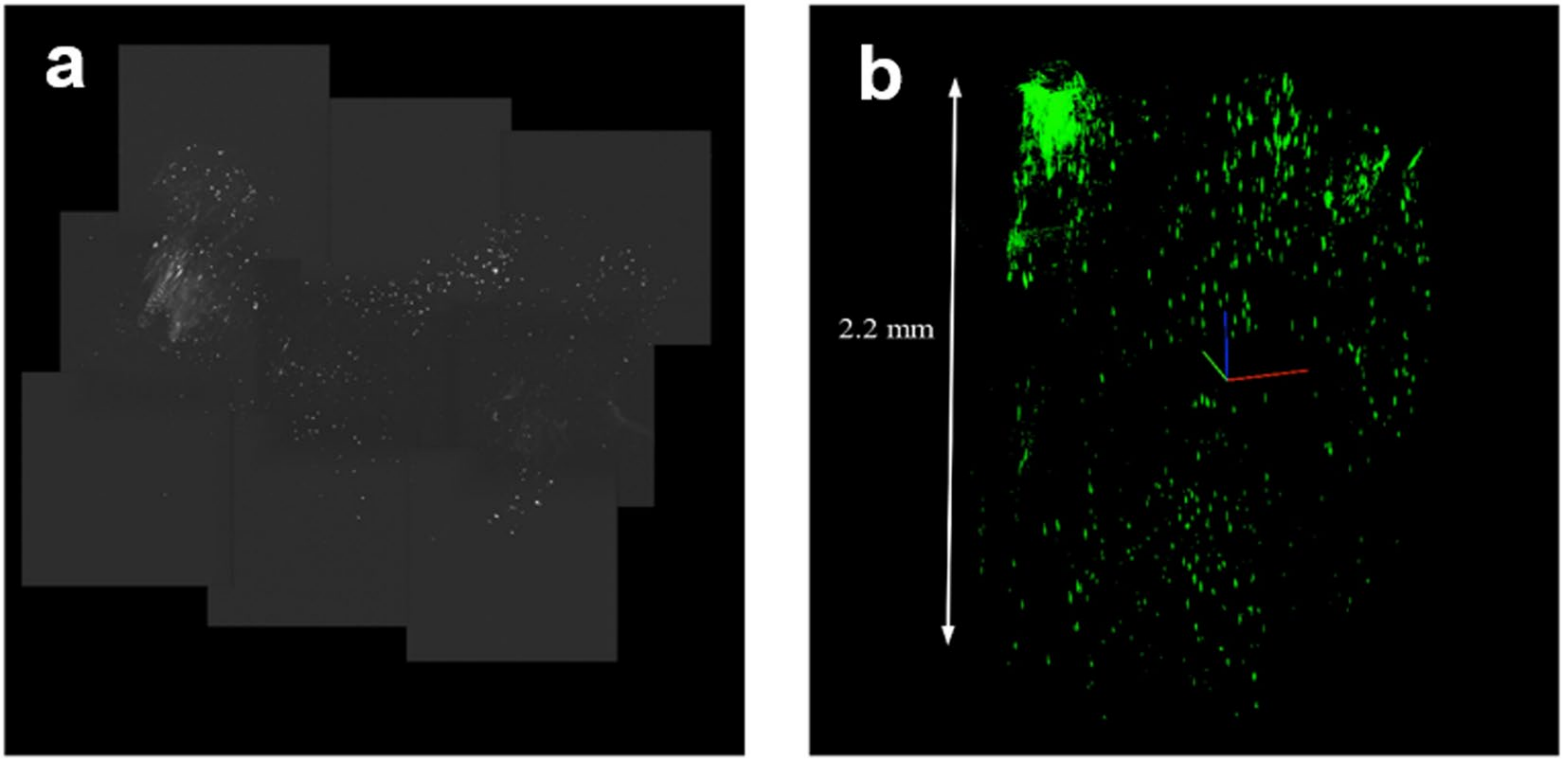
The prototype H^2^L^2^-CFM microscope was used to examine a cleared rat brain through the hippocampus and parietal cortex. (**a**) shows the maximum projection image of the cleared rat brain following immunohistochemistry for Arc protein, imaged over a depth of 2.2 mm in increments of 3 μm steps. (**b**) is a 3D reconstruction of the images shown in (**a**). The hippocampus can be observed clearly as the dense green area of this image (top left), corresponding to region that is also shown in Fig. 10(a). The parietal cortex is the region to the right of the hippocampus in (**b**), which corresponds to the cortical region shown in Fig. 10(c).

We have also begun to develop immunohistochemical methods to visualize proteins that reflect neuron activity throughout the rat brain. Immunohistochemistry for Arc protein was performed on a brain that was cleared using the passive CLARITY method^15^ followed by a fluorescein tyramide signal amplification reaction procedure. The brain was then mounted in an agarose block and immersed in 85% glycerol for imaging. Figure 11(a and b) show images of the cleared rat brain labeled with Arc protein, including the hippocampus and overlying parietal cortex, previously shown in Fig. 10(a) and (b), respectively. Figure 11(a) is a collage of images across a depth of 2.2mm  using the H^2^L^2^-CFM microscope. Figure 11(b) shows the 3D reconstruction of the images shown in (a).

## Discussion

An H^2^L^2^-CFM designed specifically for whole brain imaging of cleared rat brains is demonstrated. This microscope utilizes a custom 0.5 NA objective designed for a 3 mm × 3 mm FOV and 12 mm working distance. It uses 9 beams to simultaneously scan the FOV in 9 adjacent regions simultaneously to decrease the acquisition time by a factor of 9.

The H^2^L^2^-CFM is assembled and tested. Fluorescent microspheres dispersed in a volume of PDMS showed the working distance is at least 10 mm in depth. A measurement of 200 nm fluorescent microspheres is used to estimate the PSF of the microscope in all 9 channels. The FWHM of the estimated PSFs are 555 nm ± 88 nm. Due to the large field of view, long working distance, and high NA of this confocal fluorescence microscope, H^2^L^2^-CFM is an excellent candidate for imaging large volumes of optically cleared brains. We have demonstrated that cleared rat brain tissue can be imaged with the H^2^L^2^-CFM microscope as shown in Fig. 11. Future experiments will use this microscope in conjunction with improved brain clarification methods to image larger volumes of optically cleared brains at subcellular resolution with reduced acquisition time, since 9 beams are used to speed up acquisition of a FOV by a factor of 9.

The concept of multi-region and multifocal scanning can be used in other imaging modalities, such as multiphoton microscopes, to achieve high speed imaging. The fluorescent signal detection for multiphoton imaging is simpler than confocal fluorescence imaging because no confocal pinhole is needed. In addition, with the advance of ultrafast fiber lasers, the implementation of multi-region and multifocal multiphoton microscope will be much easier. According to our preliminary study, the FOV can be extended beyond 12 mm × 12 mm.
